# Storage causes protein oxidation of soybean meal and affects antioxidant status, digestive performance and meat quality of broilers

**DOI:** 10.5713/ab.24.0011

**Published:** 2024-08-22

**Authors:** Peng Wang, Juanjuan Song, Mingfang Du, Chao Wen, Yanmin Zhou

**Affiliations:** 1 College of Animal Science and Technology, Nanjing Agricultural University, Nanjing 210095, China

**Keywords:** Broilers, Protein Oxidation, Storage, Soybean Meal

## Abstract

**Objective:**

This study investigated the protein oxidation of soybean meal (SBM) stored in a warehouse and the effects of SBM on growth performance, antioxidant status, digestive performance, intestinal morphology, and breast muscle quality of broilers.

**Methods:**

In total, 160 one-day-old Arbor Acres Plus broilers (half male and half female) were randomly divided into two groups with ten replicates of eight birds each: The control group was served with a basal diet including SBM stored at −20°C (FSBM), and the experimental group was served with a basal diet including SBM stored in a warehouse at room temperature for 45 days (RSBM).

**Results:**

Compared with FSBM, the protein carbonyl level in RSBM was increased, the free and total thiol levels and *in vitro* digestibility of protein were decreased. The RSBM decreased the serum glutathione (GSH) level and the hepatic total superoxide dismutase (T-SOD) activity at days 21 and 42 when compared with FSBM. Further, RSBM reduced the duodenal T-SOD activity, jejunal catalase (CAT), and T-SOD activities at day 21, and decreased the duodenal CAT and T-SOD activities, jejunal T-SOD activity, and ileal GSH level and T-SOD activity at days 21 and 42 when compared with FSBM. Besides, the trypsin activity and the ratio of villus height to crypt depth in small intestines of broilers at days 21 and 42 were reduced when fed with a RSBM-contained diet. Compared with FSBM, the 24-h drip loss, shear force, and 24- and 48-h cooking loss of breast muscle were increased of RSBM group, the opposite result was observed for muscle lightness at 48 h.

**Conclusion:**

Room temperature storage for 45 days led a protein oxidation and decreased *in vitro* digestibility in SBM, and fed RSBM impaired growth performance, antioxidant status, and meat quality, reduced trypsin activity, and affected the small intestine morphology in broilers.

## INTRODUCTION

Soybean meal (SBM), a byproduct from soybean oil extraction, is one of the main protein feeds for farm animals, with a crude protein content of 40% to 48%. SBM is widely used as a protein feed ingredient in livestock, poultry, aquaculture, and even pets. About approximately 85% of SBM is used in feeds, and poultry and swine have the largest SBM consumption owing to a well-balanced amino acid composition [1,2]. However, SBM protein quality is influenced by the type of soybean, SBM processing, transportation, and storage, which can greatly increase the oxidation sensitivity of SBM protein and cause a decrease of protein digestibility in SBM [3].

Protein oxidation is a complex series of processes in which protein molecules undergo covalent structural modifications with specific amino acid residue in the existence of free radicals or secondary oxidation products [4]. Oxidation of SBM protein is typically manifested by an increased protein carbonyl (PC) value, reduction of free thiols, and the occurrence of protein cross-linking aggregation, which results in adverse effects, such as a decrease in protein solubility [5]. Heating can result oxidation of the SBM protein, as evidenced by increased carbonyl content and decreased free and total thiols levels, exhibiting a time-dependent response to heat treatment [6]. Protein oxidation is usually due to changes in protein structure that cause changes in quality [7]. The SBM proteins can also be oxidized by a terminal product of lipid peroxidation, acrolein, leading to decreased α-helix and increased β-folding and protein aggregation, which increases when the oxidation of soy protein deepens [8]. In addition, the biological characteristics of proteins can be affected by thermal oxidation, including altered structure and loss of antioxidant capacity [9].

Storage is the inevitable process of SBM from production to utilization; the length of this time is determined by SBM futures prices and generally is within a range of one week to a few months. However, there were few studies on the effect of storage on SBM proteins and their impact on broilers. Therefore, studying the quality changes of SBM under actual storage conditions and its effects on broiler production to rationalize the use of SBM is necessary.

Protein oxidation has been shown to have adverse effects on animals. For example, studies have shown that feeding oxidized proteins can reduce antioxidant enzyme activity and redox imbalance after ingestion of oxidized soy protein in rodent animals, which was also accompanied by increased malondialdehyde contents and reactive oxygen species (ROS) levels in tissues, and decreased the activities of total superoxide dismutase (T-SOD) and catalase (CAT), and glutathione (GSH) levels [10]. Studies on domestic animals showed a negative impact on broiler immunity, growth performance, and redox status after feeding heat-treated SBM to broilers [11], and fed heated SBM can induce gut barrier damage and affect the nutrient absorption in laying hens [12].

Generally, the storage duration of SBM ranges from one week to a few months before feed manufacture. However, there are no studies on the effect of storage on SBM proteins and its effect on broilers. Therefore, it is crucial to thoroughly evaluate the oxidation of SBM protein and its impact on broiler growth under SBM storage conditions for the effective application of SBM in production.

## MATERIALS AND METHODS

### Chemicals

All the fresh SBM was produced from Yihai Kerry (Lianyungang) Grain and Oil Industry Co., Ltd (Jiangsu, China) on the day of production. The moisture, crude protein, crude fiber, and crude ash content of SBM were 12.99%, 43.13%, 6.19%, and 6.04%, respectively. All analyses were conducted in accordance with the Institute of Official Analytical Chemists. Pepsin (3,000 U/mg) and trypsin (250 NFU/mg) were obtained from Solarbio Biotechnology Co., Ltd (Beijing, China). All chemicals and reagents used in the assay section are of analytical grade.

### Storage of soybean meal

The SBM of the treatment group (RSBM) was stored in a warehouse at Xuzhou Changjiang Biotechnology Co. Ltd., Jiangsu, China, to simulate the actual storage environment of SBM completely. The average daily temperature and humidity were 22°C and 54%, respectively. In order to prevent the influence of different batches of SBM on this test, the same batch SBM was used in the control group (FSBM) and placed in a −20°C refrigerated warehouse simultaneously. The storage time was 45 days (From March 27th to May 10th). The relevant indexes were tested immediately after storage treatment and the above mentioned SBM was used for feeding trial.

### Determination of protein carbonyl value

Protein carbonyl content of RSBM and FSBM was detected by the method of 2, 4-dinitrophenylhydrazine [13]. The reacted protein solution was detected by spectrophotometer at 367 nm and the unit of the result is nanomoles of carbonyl groups per mg of soluble protein and calculated with 22,000 M^−1^ cm^−1^ as the extinction coefficient. The protein concentration in RSBM and FSBM were detected by the Bradford method and the bovine serum albumin was used as the standard [14].

### Measurements of free thiols and total thiol

The free and total thiols contents in RSBM and FSBM were detected by the Ellman’s method [15]. Solution detection wavelength was set at 412 nm and calculated with 13,600 M^−1^ cm^−1^ as the extinction coefficient. The same method was used to determine the SBM protein concentration.

### Measurements of *in vitro* digestibility of soybean meal protein

The *in vitro* determination of SBM protein was performed according to the method of Lo and Li-Chan [16] with some modifications. First, the pH of the prepared 3% SBM suspension was stabilized to 2.0. Next, 2% pepsin was added and shaken for 4 h at 39°C. The pH of the digestive juice was then changed to 7.0 and the digestion solution was incubated at 40°C for 4 h. Following the digestion experiment, precipitated the protein in the digest with 5 mL of trichloroacetic acid, and centrifuged at 10,000×*g* for 15 min to separate the precipitate and supernatant, and stored them in an ultra-low temperature refrigerator.

### Determination of amino acid contents

The 0.1 g of SBM powder was added to the ampoule, and 5 mL of 6 mol/L hydrochloric acid was added, and then filled the ampoule with a nitrogen blower. The ampoules were sealed with an explosion-proof gas cylinder and placed in an oven at 110°C for 24 h. The solution in the ampoule was diluted to 50 mL with pure water. Next, the 1 mL of the diluted solution was transferred to a 10 mL centrifuge tube and dried with a nitrogen blower. To dissolve the residue, 2 mL of hydrochloric acid with a concentration of 0.02 mol/mL was added. Then the solution was filtered through a 0.22 μm membrane and finally analyzed using an automatic amino acid analyzer (Hitachi LA-8080; Hitachi, Tokyo, Japan).

### Broilers and management

The standard of the animal experiment part of this experiment referred to the guidelines of the Institutional Animal Ethics Committee of Nanjing Agricultural University, and was strictly implemented in accordance with the regulations. The license number is SYXK-2017-0007. In total, 160 one-day-old Arbor Acres Plus broilers (half male and half female) were randomly divided into two groups with ten replicates of eight birds each: The control group was served with a basal diet including SBM stored at −20°C (FSBM), and the experimental group was served with a basal diet including SBM stored in a warehouse at room temperature for 45 days (RSBM). The animal experiment was carried out at the Baima base of Nanjing Agricultural University with a 42-d trial period. The broilers were kept in 4-tier cages (0.085 m^2^ per chicken at days 1 to 21; 0.094 m^2^ at days 22 to 42) with continuous lighting for 23 h, and each cage was equipped with 1 feeder and 2 nipple drinkers. The temperature was kept at 32°C±2°C for the first 3 days, then decreased by 1°C every two days until the temperature was 20°C. The feed intake of broilers at each stage of the experiment (1 to 21 days of age and 22 to 42 days of age) and the whole period of the experiment (1 to 42 days of age) was recorded in replicates, and the broilers were weighed in replicates on a fasting basis at the ages of 1, 21, and 42 days of age, respectively (12 hours of fasting and free-flowing water), which were used to calculate the growth performance indices such as the mean body weight, the average daily feed intake (ADFI), the average daily gross gain (ADG), and the ratio of feed to gain (F/G) of the broilers. The formulation and nutrient levels of the basal diet for broilers were formulated according to Table 1.

### Sample collection

One broiler with smooth feathers, good mental condition, and near average body weight was selected for each replicate at days 21 and 42, and 2 mL of blood was collected after 8-h fasting and the serum was prepared by centrifugation and stored frozen at −20°C. Broilers were sacrificed using the neck twisting method to isolate the liver, breast muscle, and small intestine. The tissues of the duodenum (U-shaped collaterals), jejunum (anterior 1/4), and ileum (middle 1/2) were sectioned to determine villus height (VH) and crypt depth (CD), and the mucous membrane of small intestine were collected in Eppendorf tubes then frozen in liquid nitrogen until analysis.

### Determination of antioxidant status

To measure the activity of antioxidant enzymes, the tissue or serum samples were pretreated respectively according to the kit’s requirements. The quality control and detection parameter settings of the samples all meet the product specifications. In order to measure the T-SOD activity, the xanthine oxidase method was utilized. The GSH level was measured using the 5,5′-dithiobis-(2-nitrobenzoic acid) method described by Anderson [17]. The total antioxidant capacity (T-AOC) was determined using the ferric reducing ability method, as outlined by Benzie and Strain [18]. And the CAT activity was measured using the ammonium molybdate method while glutathione peroxidase (GSH-Px) activity were measured using the dithionitrobenzoic acid method [19,20]. The measurement kits used were from Nanjing Jiancheng Institute of Bioengineering, located in Nanjing, Jiangsu, P. R. China.

### Determination of digestive enzyme activity

The chyme from the duodenum, jejunum, and ileum was pretreated according to the kit′s requirements for further detection. The trypsin, lipase, and α-amylase activity measurements were carried out using kits from Nanjing Jiancheng Institute of Bioengineering, located in Nanjing, Jiangsu, P. R. China, following the manufacturer′s instructions. Crystalline bovine serum albumin was used as a reference standard.

### Histological measurement

The samples in paraformaldehyde were ethanol-dehydrated, embedded in paraffin, and stained with hematoxylin-eosin. Slices of the small intestine were then observed under a microscope (Nikon80i; Nikon, Tokyo, Japan) and recorded. Six VH and six CD values were measured for each sample, and the mean values were counted after measurement to calculate VH/CD.

### Determination of meat quality

The right pectoral muscle of slaughtered broilers was removed at day 42, and the pH of meat was measured at 45 min, 24 h, and 48 h with FiveGo F2 (Mettler Toledo, Switzerland). The values of the lightness (L*), redness (a*), and yellowness (b*) were measured by the CR-10 colorimeter (Konica Minolta, Tokyo, Japan) at 45 min, 24 h, and 48 h. Additionally, the Meat Tenderometer (G-R Manufacturing, Trussville, AL, USA) was utilized to measure shear force [21]. A 5×3×1 cm pectoral muscle sample was weighed (m_1_), and the meat samples were sealed in a box with plastic wrap, placed in a refrigerator at 4°C, and weighed respectively after 24 and 48 h (m_2_). The drip loss rate (X_1_) was calculated with the following formula (1). A smooth surface without damage to the thoracic muscle sample piece was selected, and the surface of the tendons, membranes, and fat, weighing approximately 30 g (m_3_), was removed, put into a water bath at 80°C in a sealed bag, then removed immediately when the meat center temperature was up to 75°C in the water bath (approximately 10 min, using a thermometer for detection), and then the center temperature was cooled to 18°C below with an ice bath (approximately 20 min). The sample was removed with filter paper to dry the surface moisture after weighing m_4_, and the steaming loss rate (X_2_) was calculated with the following formula (2).


(1) $${\text{X}}_{1}=({\text{m}}_{1}-{\text{m}}_{2})/\text{mL}\times 100\%$$

(2) $${\text{X}}_{2}=({\text{m}}_{3}-{\text{m}}_{4})/{\text{m}}_{3}\times 100\%$$

### Statistical analysis

The data were analyzed using SPSS statistical software (Chicago, IL, USA) through independent samples T-test analysis. A significant difference was noted when p<0.05. Mean and standard error of the mean were utilized to express all data. The related images were created by Graphpad Prism 8.0 (GraphPad Software, San Diego, CA, USA).

## RESULTS

### Protein oxidation and amino acid changes in soybean meal after storage

**Figure 1:**
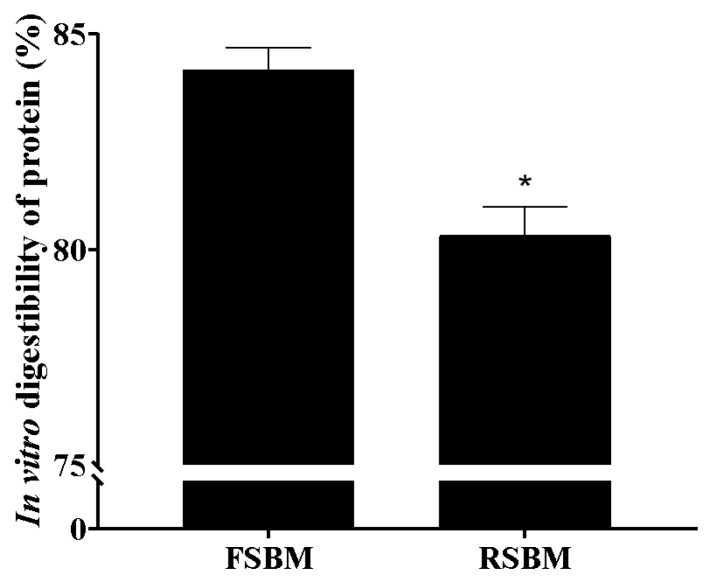
Effects of storage on the *in vitro* digestibility of proteins in soybean meal. FSBM, soybean meal frozen for 45 d; RSBM, soybean meal stored at room temperature for 45 d. * Indicates p<0.05.

Compared with FSBM (Table 2), the RSBM had a higher PC value (p<0.05), and the content of free and total thiols in RSBM was significantly reduced (p<0.05). The results presented in Figure 1 indicate that the *in vitro* crude protein digestibility of RSBM was lower compared with that of FSBM (p<0.05). Table 3 shows that the methionine content in the RSBM group was significantly lower than that in the FSBM group (p<0.05).

### Growth performance

As summarized in Table 4, birds fed diets containing RSBM tended to have lower FCR (p = 0.066). However, dietary RSBM has no changes in ADG and ADFI at days 21 and 42 (p>0.05).

### Antioxidant status in serum and tissues

The RSBM had decreased GSH levels in serum at day 21 and T-SOD activity in liver at days 21 and 42 compared with the FSBM (p<0.05) (Table 5). In addition, the CAT, GSH-Px, and T-AOC activities in the serum and liver were not altered by different treatments (p>0.05). Compared with the FSBM, feeding RSBM reduced duodenal T-SOD, jejunal CAT, and T-SOD activities and ileal GSH level at day 21 (p<0.05) (Table 6). In addition, duodenal CAT activity, T-SOD activity, jejunal T-SOD activity, ileal GSH level, and T-SOD activity were decreased by RSBM at day 42, while increased duodenal GSH-Px activity at day 42 (p<0.05).

### The activity of intestinal digestive enzymes

The RSBM had lower trypsin activity of the foregut and hindgut at days 21 and 42 compared with the FSBM (p<0.05) (Table 7). Meanwhile, duodenal *α*-amylase activity in the foregut and hindgut in the RSBM group showed a significant increase at day 42 (p<0.05). However, treatments did not alter the intestinal lipase activity of broilers at days 21 and 42 (p>0.05).

### Morphology of small intestinal mucosa

**Figure 2:**
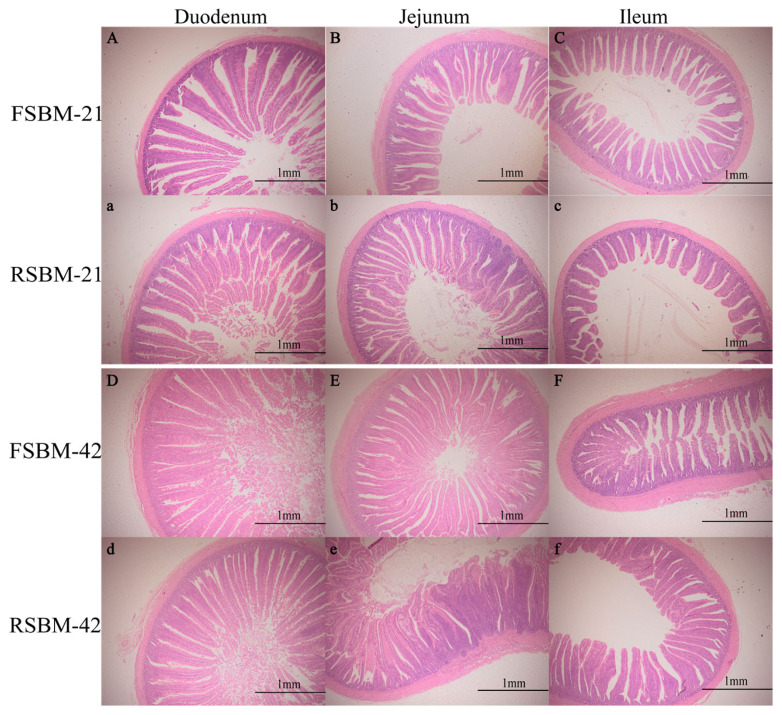
Effects of storage of soybean meal on the small intestine morphology in broilers. FSBM, soybean meal frozen for 45 d; RSBM, soybean meal stored at room temperature for 45 d.

Table 8 showed that the duodenal VH in the RSBM group at day 21 was lower than that of the FSBM group, but the CD in the jejunum and ileum was significantly increased (p<0.05), the ileal VH in the RSBM group increased significantly at day 42 compared with the FSBM group (p<0.05). Simultaneously, the ratio of VH to CD in the duodenum and jejunum significantly decreased at days 21 and 42 (p<0.05). The ratio of VH to CD in the ileum at days 21 and 42 was no significant difference (p>0.05). In addition, RSBM caused similar inflammatory phenomena in the intestinal villus at days 21 and 42 when compared with FSBM (Figure 2).

### Meat quality

Table 9 showed the effects of RSBM on the meat quality of broilers. Compared with FSBM group, broilers fed RSBM exhibited higher drip loss, cooking loss and shear force of breast muscle at 24 h postmortem and increased cooking loses of breast muscle at 48 h postmortem (p<0.05). The brightness/L* value of 48 h muscle was reduced by RSBM treatment when compared with FSBM group (p<0.05). However, there was no significant difference on pH value of breast muscle between RSBM group and FSBM group (p>0.05).

## DISCUSSION

### Effect of storage on protein oxidation of soybean meal

The PC value reflects the degree of protein oxidation, and the free amino group is a sensitive indicator of protein oxidation [13]. Previous studies showed that the heating treatment of SBM increased the PC value of protein and decreased the free and total thiol levels [6]. In this experiment, RSBM had higher PC values and lower free and total thiols than FSBM, which was similar to previous findings. The reason may be that the amino acids (Thr, Ala, Cys, Met, Lys, and His) in proteins were more susceptible to attack by ROS and generated carbonyl compounds [22]. In particular, methionine contains sulfur, which is easily oxidized by ROS in the environment, and the oxidation of amino acids may result in the formation of carbonyl compounds and consumption of free thiols between peptide chains to form cross-linked proteins [23]. Therefore, feed formulation with a balanced amino acid content is of great importance for the nutritional requirements of animals. In addition, the decrease in sulfhydryl groups is generally attributed to the combination of free thiols into disulfide bonds [24]. These results suggest that SBM proteins were oxidized during storage, and it can be assumed that the structure of the SBM protein has undergone a change, which could potentially impact the efficiency of SBM protein utilization.

### Effect of storage on *in vitro* digestibility of soybean meal protein

The *in vitro* digestibility of proteins represents the extent of SBM proteins that can be digested by the animal. The protein digestion process is essentially the hydrolysis of proteolytic sites by proteases [25]. In this study, the *in vitro* digestibility of proteins in RSBM was significantly reduced, suggesting changes in SBM proteins during storage. Lu et al [6] found that the *in vitro* digestibility of SBM protein was significantly reduced when the SBM protein was subjected to heat-induced oxidation. Long et al [26] found a significant negative correlation between β-folding of feed proteins and their *in vitro* digestibility. Zhao et al [27] found that soybean proteins were oxidised with changes in their secondary structure, which also resulted in a reduction in digestibility. Through the above studies, it can be found that due to oxidation of proteins, the side chain amino acids may be modified, on the one hand, the original amino acids that can be recognised by proteases are lost, on the other hand, due to the changes in the secondary structure of proteins caused by oxidation, such as internal folding and intermolecular cross-linking, more enzyme digestion sites are encapsulated, which leads to a decrease in the enzymatic efficiency of proteases, and the number of enzyme digestion limits is also reduced [11,24].

### Effects of stored soybean meal on the antioxidant properties of broiler tissues

Feeding oxidized proteins effects animal growth and the antioxidant status of the organism [3]. Lu et al [6] found that fed heated-induced SBM significantly increased FCR in broilers, however, there was a tendency for the RSBM group to reduce ADG and increase FCR in broilers compared with FSBM in this study. The inconsistency in the above results may be due to the difference in the degree of protein oxidation in the SBM. Fed RSBM diets reduced serum GSH content, liver T-SOD activity, and intestinal T-SOD, CAT, and GSH-Px activities. Similar results were obtained in previous studies in broilers [6,10]. there was a relevant study which demonstrated that the mice would suffer oxidative stress after being fed oxidized casein as the basal protein for 10 weeks, as evidenced by an increase in PC value, advanced oxidation protein products, dityrosine, lipid peroxidation, and ROS levels in the liver, kidney, and serum [28], and a similar report that changes in the physical and chemical properties of soy protein can induce the production of ROS in mice [11]. Those studies the presented that the antioxidant capacity of cells is not sufficient to resist the oxidative damage caused by oxidized proteins, and severe and prolonged oxidative stress depletes antioxidants, resulting in lower antioxidant levels [29]. Some studies have shown that it may also be related to a decrease in Nrf2 mRNA expression level, which reduces the antioxidant level [6].

### Effects of stored soybean meal on intestinal digestive performance of broilers

Trypsin is the predominant enzyme that digests intestinal proteins. Highly cross-linked aggregated proteins are exposed to fewer enzymatic sites and cannot be digested effectively by trypsin, similar to the significantly lower *in vitro* digestibility of oxidized proteins in SBM. The trypsin activity in the small intestinal of broilers was significantly reduced after feeding oxidized soy protein isolate [11], which is consistent with previous study that feeding oxidated SBM reduced jejunal trypsin activity in broilers [6]. The decreased trypsin activity might be due to poor development of the small intestine, which prevents the pancreas from being fully functional in secreting digestive enzymes. Fewer sites of the oxidized protein exposed to trypsin digestion may be another reason.

### Effects of stored soybean meal on intestinal morphology of broilers

The health of an animal’s gut is critical to the digestion and absorption of nutrients [30]. Oxidative stress in the body is a potential poses health threat to chickens [31]. Zhang et al [32] found that ingestion of oxidised soy proteins causes oxidative stress in the broiler gut and negatively affects villus, which may be one of the reasons why significantly similar inflammatory changes occurred in the jejunum of broilers in the RSBM group compared with the FSBM group in this study. There was a study that showed the oxidation in SBM protein caused a decrease in VH and increased CD of broilers [6], which experimental results are consistent with the results of this study. Frame et al [33] found that intestinal VH showed a negative linear relationship with PC value, which may be due to intestinal-related inflammation triggered by the oxidation of protein. Reduced intestinal antioxidant levels in broilers may have contributed to the reduced VH, but the exact reasons for this need to be explored in studies.

### Effect of stored soybean meal on meat quality of broilers

Drip loss and cooking loss are indicators of meat water-holding capacity, they are very important in the evaluation of broiler meat quality [34]. Previous reports have demonstrated/shown that the water-holding capacity of muscle protein could be decreased under free radical attack and the muscle pH would be affected by muscle glycogen content or glycolysis rate [35,36]. In this experiment, increased drip loss and cooking loss in RSBM suggested that protein oxidation in SBM may reduce the water-holding capacity of muscle. Shear force represents muscle tenderness, to a certain extent, the smaller the muscle shear force, the better the muscle taste [37], and shear force is associated with collagen content [36]. In this study, increased shear force was found in RSBM group. There was a study reported that dietary oxidated protein may alter the Nrf2-ARE gene pathway, reducing its antioxidant enzyme activity and causing damage to the organism, which would induce oxidative injury of muscle [28], Therefore, it was hypothesized that the oxidation factors within the chicken muscle of the RSBM group accelerated protein denaturation, aggregation, and cross-linking, leading to increased shear force, thereby reducing muscle quality in this study. However, the mechanisms that caused oxidative stress have not yet been elucidated.

## CONCLUSION

In summary, room temperature storage can cause oxidation of proteins in SBM and result in decreased protein *in vitro* digestibility. Diet with RSBM may negatively affect growth performance, antioxidant status, digestibility, meat quality and intestinal morphology due to protein oxidation. However, there are few reports about the protein oxidation of SBM during storage and the mechanism of protein oxidation affecting broilers, which will be the focus in SBM of future work.

## Figures and Tables

**Table 1:** Composition and nutrient levels of the basal diets (g/kg)

Items	1 to 21 d		22 to 42 d	
	
	FSBM^1)^	RSBM^2)^	FSBM^1)^	RSBM^2)^
Ingredients				
Corn	563.20	563.20	620	620
SBM (Stored at −20°C)	348	-	292	-
SBM (Stored at room temperature)	-	348	-	292
Soybean oil	40	40	44	44
Limestone	15	15	13	13
Dicalcium phosphate	18	18	16	16
L-Lysine	1	1	1	1
DL-Methionine	1.60	1.60	1	1
Sodium chloride	3.20	3.20	3	3
Premix^3)^	10	10	10	10
Calculated nutrient levels				
Apparent metabolizable energy (MJ/kg)	12.88	12.88	13.14	13.14
Crude protein	218	218	195	195
Calcium	10.70	10.70	9.30	9.30
Available phosphorus	4.40	4.40	4.00	4.00
Lysine	11.40	11.40	10.00	10.00
Methionine	5.00	5.00	4.10	4.10
Methionine+cystine	8.60	8.60	7.40	7.40
Analyzed nutrient levels				
Gross energy (MJ/kg)	14.33	14.33	16.17	16.17
Crude protein	213.3	213.3	194.9	194.9
Calcium	10	10	9.8	9.8
Total phosphorus	6.9	6.9	6.2	6.2
Lysine	11.21	11.21	10.4	10.4
Methionine	4.8	4.8	4.1	4.1

1) FSBM, soybean meal frozen for 45 d.

2) RSBM, soybean meal stored at room temperature for 45 d.

3) Premix provided per kilogram of diets: vitamin A (transretinyl acetate), 10,000 IU; vitamin D_3_ (cholecalciferol), 3,000 IU; vitamin E (all-rac-α-tocopherol), 30 IU; menadione, 1.3 mg; thiamin, 2.2 mg; riboflavin, 8 mg; nicotinamide, 40 mg; choline chloride, 600 mg; calcium pantothenate, 10 mg; pyridoxine·HCl, 4 mg; biotin, 0.04 mg; folic acid, 1 mg; vitamin B_12_ (cobalamin), 0.013 mg; Fe (from ferrous sulfate), 80 mg; Cu (from copper sulphate), 8.0 mg; Mn (from manganese sulphate), 110 mg; Zn (from zinc oxide), 60 mg; I (from calcium iodate), 1.1 mg; Se (from sodium selenite), 0.3 mg.

**Table 2:** Effects of storage on the oxidation degree of soybean meal

Items	Treatment^1)^		p-value

	FSBM	RSBM	
Protein carbonyl (nmol/mg protein)	6.89±0.16	8.53±0.09	<0.001
Free thiols (nmol/mg protein)	8.24±0.06	6.75±0.08	<0.001
Total thiol (nmol/mg protein)	145.37±3.18	116.08±1.99	<0.001

1) FSBM, soybean meal frozen for 45 d; RSBM, soybean meal stored at room temperature for 45 d.

**Table 3:** Effect of storage on amino acid content of soybean meal (%)

Items	Treatment^1)^		p-value

	FSBM	RSBM	
Asp	4.85±0.03	4.81±0.04	0.483
Thr	1.63±0.01	1.63±0.01	0.987
Ser	1.83±0.02	1.86±0.02	0.254
Glu	7.64±0.04	7.65±0.07	0.924
Gly	1.87±0.01	1.86±0.02	0.772
Ala	1.96±0.01	1.94±0.02	0.364
Cys	0.34±0.01	0.33±0.01	0.255
Val	2.21±0.05	2.18±0.03	0.237
Met	0.40±0.01	0.36±0.01	0.005
Ile	2.10±0.01	2.07±0.02	0.206
Leu	3.48±0.02	3.44±0.03	0.398
Tyr	1.47±0.01	1.49±0.02	0.249
Phe	2.28±0.01	2.26±0.02	0.553
Lys	2.82±0.02	2.80±0.02	0.532
His	1.14±0.01	1.14±0.01	0.590
Arg	3.32±0.02	3.30±0.03	0.541

1) FSBM, soybean meal frozen for 45 d; RSBM, soybean meal stored at room temperature for 45 d.

**Table 4:** Effects of stored soybean meal on the growth performance of broilers

Items	Treatment^1)^		p-value

	FSBM	RSBM	
1 to 21 days			
ADG (g/d/bird)	38.48±0.75	35.94±0.68	0.093
ADFI (g/d/bird)	51.56±1.26	49.96±1.23	0.339
FCR (g:g)	1.34±0.01	1.39±0.02	0.066
22 to 42 days			
ADG (g/d/bird)	47.40±1.85	44.83±1.64	0.313
ADFI (g/d/bird)	100.94±1.42	98.08±1.50	0.184
FCR (g:g)	2.16±0.09	2.21±0.08	0.657
1 to 42 days			
ADG (g/d/bird)	44.94±0.98	42.46±1.05	0.100
ADFI (g/d/bird)	76.25±1.02	74.02±1.08	0.151
FCR (g:g)	1.70±0.03	1.74±0.03	0.301

ADG, average daily gain; ADFI, average daily feed intake; FCR, feed conversion ratio.

1) FSBM, soybean meal frozen for 45 d; RSBM, soybean meal stored at room temperature for 45 d.

**Table 5:** Effects of stored soybean meal on the antioxidant status of the serum and liver

Items	Treatment^1)^		p-value

	FSBM	RSBM	
21 days			
Serum			
CAT (U/mL)	3.28±0.73	4.71±1.97	0.481
GSH (μmol/mL)	80.41±12.38	45.19±5.68	0.039
T-SOD (U/mL)	214.69±14.79	224.64±15.25	0.643
GSH-Px (U/mL)	564.58±7.18	536.04±21.33	0.240
T-AOC (U/mL)	3.14±0.13	3.28±0.15	0.492
Liver			
CAT (U/mg protein)	15.73±1.27	13.54±1.68	0.327
GSH (mg/g protein)	17.16±3.19	22.12±5.58	0.421
T-SOD (U/mg protein)	122.96±2.98	108.76±5.19	0.024
GSH-Px (U/mg protein)	15.39±1.34	15.84±1.24	0.814
T-AOC (U/mL protein)	3.48±0.79	2.21±0.08	0.157
42 days			
Serum			
CAT (U/mL)	2.52±0.63	3.14±0.92	0.589
GSH (μmol/mL)	35.41±3.32	30.06±2.01	0.208
T-SOD (U/mL)	208.22±16.83	202.64±17.99	0.822
GSH-Px (U/mL)	505.38±21.02	468.78±18.03	0.226
T-AOC (U/mL)	3.29±0.26	3.69±0.11	0.210
Liver			
CAT (U/mg protein)	23.31±3.77	13.65±4.66	0.138
GSH (mg/g protein)	13.52±1.72	17.23±4.20	0.384
T-SOD (U/mg protein)	211.64±5.23	194.58±3.80	0.016
GSH-Px (U/mg protein)	23.89±1.43	23.54±1.75	0.881
T-AOC (U/mL protein)	3.08±0.68	2.05±0.08	0.181

CAT, catalase; GSH, reduced form of glutathione; T-SOD, total superoxide dismutase; GSH-Px, glutathione peroxidase; T-AOC, total antioxidant capacity.

1) FSBM, soybean meal frozen for 45 d; RSBM, soybean meal stored at room temperature for 45 d.

**Table 6:** Effects of stored soybean meal on the antioxidant status of the small intestine

Items	Treatment^1)^		p-value

	FSBM	RSBM	
21 days			
Duodenum			
CAT (U/mg protein)	13.03±2.57	7.07±1.25	0.064
GSH (mg/g protein)	42.52±4.39	38.87±4.80	0.588
T-SOD (U/mg protein)	141.90±2.80	127.82±3.88	0.006
GSH-Px (U/mg protein)	4.99±0.47	6.29±0.59	0.125
T-AOC (U/mL protein)	1.37±0.27	1.86±0.35	0.309
Jejunum			
CAT (U/mg protein)	29.45±2.31	11.13±1.51	<0.001
GSH (mg/g protein)	41.75±4.36	31.39±1.59	0.055
T-SOD (U/mg protein)	133.79±3.61	108.94±1.57	<0.001
GSH-Px (U/mg protein)	6.73±1.12	4.13±0.48	0.550
T-AOC (U/mL protein)	0.77±0.09	1.08±0.23	0.254
Ileum			
CAT (U/mg protein)	13.33±4.71	14.82±2.46	0.784
GSH (mg/g protein)	34.77±4.91	22.58±1.07	0.041
T-SOD (U/mg protein)	175.99±10.82	160.14±2.54	0.204
GSH-Px (U/mg protein)	8.80±0.89	8.90±0.80	0.941
T-AOC (U/mL protein)	1.15±0.24	1.28±0.04	0.597
42 days			
Duodenum			
CAT (U/mg protein)	46.77±9.12	16.54±2.10	0.009
GSH (mg/g protein)	19.90±2.20	25.58±2.71	0.124
T-SOD (U/mg protein)	186.93±1.86	113.32±1.98	<0.001
GSH-Px (U/mg protein)	5.03±0.75	9.26±0.46	<0.001
T-AOC (U/mL protein)	1.16±0.09	0.85±0.17	0.170
Jejunum			
CAT (U/mg protein)	47.60±7.57	30.97±5.81	0.094
GSH (mg/g protein)	45.29±4.85	44.77±4.33	0.939
T-SOD (U/mg protein)	253.17±2.20	233.09±3.66	<0.001
GSH-Px (U/mg protein)	9.61±2.53	9.40±0.98	0.940
T-AOC (U/mL protein)	1.01±0.29	0.91±0.11	0.756
Ileum			
CAT (U/mg protein)	40.55±6.78	51.20±8.94	0.365
GSH (mg/g protein)	24.13±1.22	18.39±0.93	0.020
T-SOD (U/mg protein)	131.68±2.66	101.44±2.80	<0.001
GSH-Px (U/mg protein)	6.69±0.55	5.42±1.01	0.300
T-AOC (U/mL protein)	0.97±0.23	0.97±0.16	0.986

CAT, catalase; GSH, reduced form of glutathione; T-SOD, total superoxide dismutase; GSH-Px, glutathione peroxidase; T-AOC, total antioxidant capacity.

1) FSBM, soybean meal frozen for 45 d; RSBM, soybean meal stored at room temperature for 45 d.

**Table 7:** Effects of stored soybean meal on the digestive ability of the small intestine in broilers

Items^1)^	Treatment^2)^		p-value

	FSBM	RSBM	
21 days			
Trypsin (U/mg protein)			
Foregut	12.45±0.34	10.14±0.39	<0.001
Hindgut	11.73±0.16	7.30±0.18	<0.001
α-Amylase (U/mg protein)			
Foregut	63.67±14.78	58.95±13.67	0.680
Hindgut	24.77±7.12	16.13±5.98	0.071
Lipase (U/g protein)			
Foregut	226.37±13.50	238.78±14.93	0.549
Hindgut	124.57±12.84	99.26±6.80	0.112
42 days			
Trypsin (U/mg protein)			
Foregut	2.61±0.07	2.06±0.15	0.010
Hindgut	8.33±0.72	5.17±0.42	0.003
α-Amylase (U/mg protein)			
Foregut	1.82±0.12	2.81±0.32	0.029
Hindgut	8.91±1.01	17.22±2.09	0.016
Lipase (U/g protein)			
Foregut	30.15±1.38	36.76±3.97	0.200
Hindgut	88.36±8.72	64.69±6.66	0.053

1) The foregut includes the duodenum and jejunum, and the hindgut includes the ileum.

2) FSBM, soybean meal frozen for 45 d; RSBM, soybean meal stored at room temperature for 45 d.

**Table 8:** Effects of stored soybean meal on the morphology of the small intestinal mucosa of broilers

Items	Treatment^1)^		p-value

	FSBM	RSBM	
21 days			
Villus height (μm)			
Duodenum	1,570.67±45.02	1,514.85±38.65	<0.001
Jejunum	1,072.38±20.30	1,120.57±22.53	0.141
Ileum	600.10±13.01	638.26±14.09	0.201
Crypt depth (μm)			
Duodenum	133.04±3.86	152.56±6.79	<0.001
Jejunum	122.71±5.63	148.21±4.72	<0.001
Ileum	100.22±5.08	110.29±3.74	0.215
VH/CD^2)^			
Duodenum	11.17±0.71	9.40±0.70	<0.001
Jejunum	9.13±0.37	7.86±0.24	0.003
Ileum	6.19±0.37	6.04±0.18	0.715
42 days			
Villus height (μm)			
Duodenum	1,691.87±36.39	1,603.14±30.94	0.066
Jejunum	1,425.75±35.29	1,226.11±25.03	0.003
Ileum	728.75±16.11	844.09±24.30	<0.001
Crypt depth (μm)			
Duodenum	169.00±6.02	177.24±7.73	0.415
Jejunum	150.86±7.07	168.24±9.56	0.196
Ileum	117.50±3.57	121.26±5.22	0.554
VH/CD^2)^			
Duodenum	10.39±0.43	9.54±0.38	0.014
Jejunum	9.55±0.60	7.41±0.44	0.049
Ileum	6.50±0.22	6.59±0.20	0.786

1) FSBM, soybean meal frozen for 45 d; RSBM, soybean meal stored at room temperature for 45 d.

2) VH/CD, villus height/crypt depth.

**Table 9:** Effects of stored soybean meal on pH and meat color of the breast muscle of broilers at day 42

Items^2)^	Treatment^1)^		p-value

	FSBM	RSBM	
Drip loss at 24 h (%)	4.96±0.14	6.93±0.81	0.027
Cooking loss at 24 h (%)	17.95±0.33	19.85±0.49	0.036
Shear force at 24 h (Kgf)	1.74±0.15	2.45±0.14	0.003
Drip loss at 48 h (%)	6.48±0.34	7.77±0.78	0.149
Cooking loss at 48 h (%)	19.62±0.69	21.46±0.41	0.046
Shear force at 48 h (kgf)	2.25±0.34	2.80±0.28	0.232
L*_45 min_	47.58±0.87	45.93±0.64	0.144
a*_45 min_	2.05±0.11	1.72±0.19	0.152
b*_45 min_	10.15±0.71	10.40±0.65	0.799
L*_24 h_	52.84±0.60	51.40±1.13	0.275
a*_24 h_	3.16±0.27	3.61±0.33	0.308
b*_24 h_	12.26±0.76	12.63±0.34	0.657
L*_48 h_	53.42±0.43	51.78±0.65	0.049
a*_48 h_	3.57±0.21	3.59±0.21	0.947
b*_48 h_	13.44±0.74	13.91±0.54	0.613
pH_45 min_	6.31±0.06	6.25±0.04	0.441
pH_24 h_	5.80±0.05	5.75±0.03	0.364
pH_48 h_	5.78±0.03	5.74±0.03	0.313

1) FSBM, soybean meal frozen for 45 d; RSBM, soybean meal stored at room temperature for 45 d.

2) L*, brightness; a*, redness; b*, yellowness.
